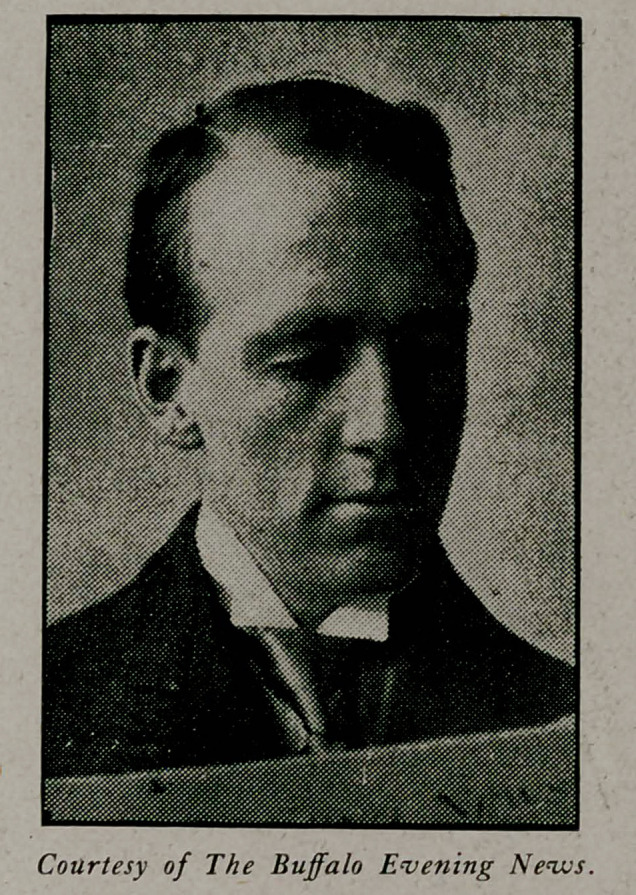# Dr. Alexander M. Troup

**Published:** 1913-10

**Authors:** 


Dr. Alexander M. Troup, Buffalo, 1900, of Holland, N. Y., was killed by the skidding and overturning of his automobile while on his way to a patient August 24. Death was instantaneous. He was born in Buffalo February 10, 1878, and was educated at the Buffalo Grammar and High Schools. He moved to Holland in 1908.

## Figures and Tables

**Figure f1:**